# [8-(4-Chloro­benzo­yl)-2,7-dimeth­oxy­naphthalen-1-yl](2,4,6-trimethyl­phen­yl)methanone

**DOI:** 10.1107/S1600536812008112

**Published:** 2012-02-29

**Authors:** Toyokazu Muto, Kosuke Sasagawa, Akiko Okamoto, Hideaki Oike, Noriyuki Yonezawa

**Affiliations:** a Department of Organic and Polymer Materials Chemistry, Tokyo University of Agriculture and Technology, 2-24-16 Naka-machi, Koganei, Tokyo 184-8588, Japan

## Abstract

In the title compound, C_29_H_25_ClO_4_, the dihedral angle between the benzene rings of the 2,4,6-trimethyl­benzoyl group and the 4-chloro­benzoyl group is 65.19 (9)°. The dihedral angles between the naphthalene ring system and the benzene rings of the 2,4,6-trimethyl­benzoyl group and the 4-chloro­benzoyl group are 85.66 (8) and 69.48 (8)°, respectively. In the crystal, two types of inter­molecular C—H⋯O inter­actions and an intra­molecular C—H⋯O inter­action are observed. Moreover, there is a short intra­molecular C=O⋯C=O contact of 2.614 (2) Å between the benzoyl substituents.

## Related literature

For electrophilic aromatic substitution of naphthalene derivatives, see: Okamoto & Yonezawa (2009 ▶); Okamoto *et al.* (2011 ▶). For the structures of closely related compounds, see: Mitsui *et al.* (2008 ▶); Muto *et al.* (2011*a* ▶,*b* ▶, 2012 ▶); Nakaema *et al.* (2007 ▶).
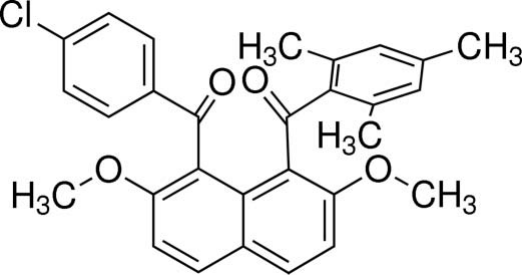


## Experimental

### 

#### Crystal data

C_29_H_25_ClO_4_
*M* *_r_* = 472.94
Monoclinic, 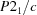

*a* = 11.6017 (2) Å
*b* = 12.3381 (2) Å
*c* = 16.2825 (3) Å
β = 90.503 (1)°
*V* = 2330.64 (7) Å^3^
*Z* = 4
Cu *K*α radiation
μ = 1.73 mm^−1^
*T* = 193 K
0.30 × 0.20 × 0.10 mm

#### Data collection

Rigaku R-AXIS RAPID diffractometer
Absorption correction: numerical (*NUMABS*; Higashi, 1999 ▶) *T*_min_ = 0.625, *T*_max_ = 0.846
40504 measured reflections
4266 independent reflections
3197 reflections with *I* > 2σ(*I*)
*R*_int_ = 0.054

#### Refinement

*R*[*F* ^2^ > 2σ(*F* ^2^)] = 0.042
*wR*(*F* ^2^) = 0.131
*S* = 1.15
4266 reflections
313 parameters
H-atom parameters constrained
Δρ_max_ = 0.21 e Å^−3^
Δρ_min_ = −0.24 e Å^−3^

### 

Data collection: *PROCESS-AUTO* (Rigaku, 1998 ▶); cell refinement: *PROCESS-AUTO*; data reduction: *CrystalStructure* (Rigaku/MSC, 2004 ▶); program(s) used to solve structure: *SIR2004* (Burla *et al.*, 2005 ▶); program(s) used to refine structure: *SHELXL97* (Sheldrick, 2008 ▶); molecular graphics: *ORTEPIII* (Burnett & Johnson, 1996 ▶); software used to prepare material for publication: *SHELXL97*.

## Supplementary Material

Crystal structure: contains datablock(s) I, global. DOI: 10.1107/S1600536812008112/gk2460sup1.cif

Structure factors: contains datablock(s) I. DOI: 10.1107/S1600536812008112/gk2460Isup2.hkl

Supplementary material file. DOI: 10.1107/S1600536812008112/gk2460Isup3.cml

Additional supplementary materials:  crystallographic information; 3D view; checkCIF report

## Figures and Tables

**Table 1:** Hydrogen-bond geometry (Å, °)

*D*—H⋯*A*	*D*—H	H⋯*A*	*D*⋯*A*	*D*—H⋯*A*
C23—H23⋯O2^i^	0.95	2.54	3.413 (2)	154
C28—H28*A*⋯O1^ii^	0.98	2.56	3.418 (3)	147
C29—H29*B*⋯O2	0.98	2.42	3.349 (3)	157

Symmetry codes: (i) 
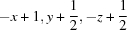
; (ii) 
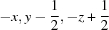
.

**Fig. 1.**
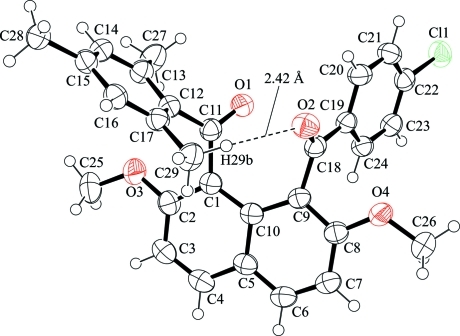
Molecular structure with displacement ellipsoids at 50% probability and a weak intramolecular C—H···O interactions.

**Fig. 2.**
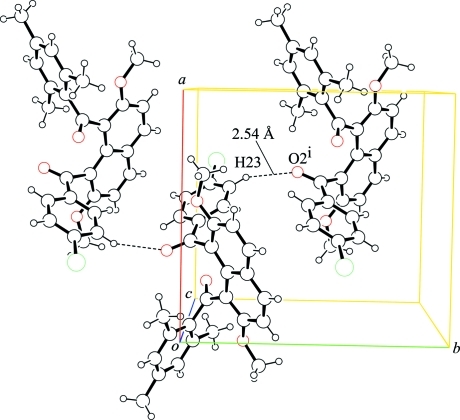
Intermolecular C23—H23···O2i interactions, viewed along the c axis [symmetry code: (i) –x + 1, y + 1/2, -z + 1/2].

**Fig. 3.**
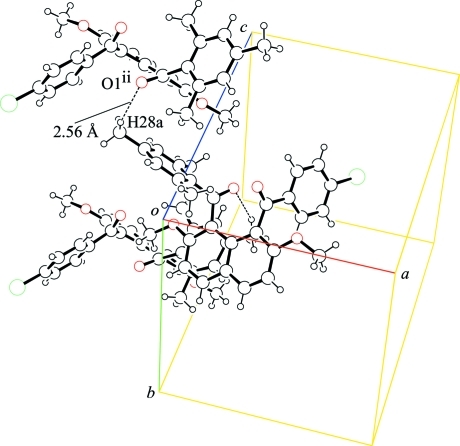
A packing diagram of the title compound, showing intermolecular C28—H28a···O1 ii interactions [symmetry code: (ii) –x, y - 1/2, -z + 1/2].

## supplementary crystallographic information

### Comment

In the course of our study on electrophilic aromatic aroylation of 2,7-dimethoxynaphthalene, *peri*-aroylnaphthalene compounds have proven to be formed regioselectively with the aid of suitable acidic mediators (Okamoto & Yonezawa, 2009; Okamoto, Mitsui *et al.*, 2011). Recently, we have reported the crystal structures of several 1,8-diaroylated naphthalene analogues exemplified by 1,8-bis(4-chlorobenzoyl)-2,7-dimethoxynaphthalene (Nakaema *et al.*, 2007) and 1,8-bis(2,4,6-trimethylbenzoyl)-2,7-dimethoxynaphthalene (Muto *et al.*, 2012). The aroyl groups at the 1,8-positions of the naphthalene rings in these compounds are connected to the naphthalene rings in an almost perpendicular fashion. Besides, the crystal structures of 1-monoaroylated naphthalene derivatives and the *β*-isomers of 3-monoaroylated derivatives have been also clarified such as 1-(4-chlorobenzoyl)-2,7-dimethoxynaphthalene (Mitsui *et al.*, 2008), (2,7-dimethoxynaphthalen-1-yl)(2,4,6-trimethylphenyl)methanone (Muto *et al.*, 2011*a*) and (3,6-dimethoxynaphthalen-2-yl)(2,4,6-trimethylphenyl)methanone (Muto *et al.*, 2011*b*).

As a part of our continuing study on the molecular structures of these homologous molecules, the crystal structure of title compound, unsymmetrical *peri*-substituted naphthalene bearing 2,4,6-trimethylbenzoyl group and 4-chlorobenzoyl group, is discussed in this report.

The molecular structure of the title compound is displayed in Fig. 1. The 2,4,6-trimethylphenyl group and 4-chlorophenyl group are out of the plane of the naphthalene ring. Two kinds of phenyl rings make different dihedral angles with the naphthalene ring system, *i.e.*, the dihedral angle between the best planes of the 2,4,6-trimethylphenyl ring (C12—C17) and the naphthalene ring (C1—C10) is 85.66 (8)°, whereas, that between the best planes of the 4-chlorophenyl ring (C19—C24) and the naphthalene ring (C1—C10) is 69.48 (8)°. Each of dihedral angles is similar to that of the corresponding symmetric 1,8-diaroylnaphthalene. The dihedral angles between the best planes of the 2,4,6-trimethylphenyl rings and the naphthalene ring of 1,8-bis(2,4,6-trimethylbenzoyl)-2,7-dimethoxynaphthalene are 81.58 (5) and 84.92 (6)° (Muto *et al.*, 2012). In addition, the dihedral angles between the best planes of the 4-chlorophenyl rings and the naphthalene ring of 1,8-bis(4-chlorobenzoyl)-2,7-dimethoxynaphthalene are 71.55 (7) and 71.98 (7)° (Nakaema *et al.*, 2007).

Besides, an intramolecular C—H···O interaction between methyl group and carbonyl group is observed (C29—H29*b*···O2 = 2.42 Å; Fig. 1 and Table 1).

The crystal packing is additionally stabilized by an intermolecular C—H···O interaction between the oxygen atom (O2) of the carbonyl group and one hydrogen atom (H23) on 4-chlorophenyl group of the adjacent molecule along the *b* axis (C23—H23···O2^i^; Fig. 2 and Table 1). Furthermore, an intermolecular C—H···O hydrogen bonding between the oxygen atom (O1) of the carbonyl group and one hydrogen atom (H28*a*) of the 4-methyl group on 2,4,6-trimethylphenyl ring of the adjacent molecule along the *b* axis is observed (C28—H28*a*···O1 ^ii^; Fig. 3 and Table 1).

### Experimental

To a 10 ml flask, 4-chlorobenzoyl chloride (0.40 mmol, 0.070 g), titanium chloride (1.20 mmol, 0.228 g) and methylene chloride (0.50 ml) were placed and stirred at rt. To the reaction mixture thus obtained, 1-(2,4,6-trimethylbenzoyl)-2,7-dimethoxynaphthalene (0.20 mmol, 0.067 g) was added. After the reaction mixture was stirred at rt for 9 h, it was poured into ice-cold water (10 ml). The aqueous layer was extracted with CHCl_3_ (10 ml × 3). The combined extracts were washed with 2 *M* aqueous NaOH followed by washing with brine. The organic layers thus obtained were dried over anhydrous MgSO_4_. The solvent was removed under reduced pressure to give cake (quant.). The crude product was purified by recrystallization from hexane and CHCl_3_ (yield 2%).

^1^H NMR δ (300 MHz, CDCl_3_); 2.16 (6*H*, s), 2.25 (3*H*, s), 3.47 (3*H*, s), 3.68 (3*H*, s), 6.77 (2*H*, s), 7.10 (1*H*, d, *J* = 9.0 Hz), 7.23 (1*H*, d, *J* = 9.3 Hz), 7.34 (2*H*, d, *J* = 8.7 Hz), 7.74 (2*H*, d, *J* = 8.7 Hz), 7.92 (1*H*, d, *J* = 8.7 Hz), 7.94 (1*H*, d, *J* = 9.0 Hz) p.p.m..

^13^C NMR δ (125 MHz, CDCl_3_); 21.11, 21.35, 56.27, 56.83, 111.13, 112.39, 121.13, 124.87, 125.72, 128.13, 129.26, 129.57, 130.13, 132.43, 133.27, 137.88, 138.37, 138.57, 139.21, 157.20, 157.94, 195.83, 199.69 p.p.m..

IR (KBr); 1656 (C=O), 1607, 1514, 1457(Ar, naphthalene), 1271 (=C—O—C) cm^-1^.

HRMS (*m/z*); [*M* + Na]^+^ Calcd for C_29_H_25_ClO_4_Na, 495.1370; found, 495.1339.

m.p. = 503.0–505.0 K.

### Refinement

All H atoms were found in a difference map and were subsequently refined as riding atoms, with C—H = 0.95 (aromatic) and 0.98 (methyl) Å, and with *U*_ĩso_(H) = 1.2 *U*_eq_(C).

### Crystal data

**Table:**

C_29_H_25_ClO_4_	*F*(000) = 992
*M**_r_* = 472.94	*D*_x_ = 1.348 Mg m^−^^3^
Monoclinic, *P*2_1_/*c*	Cu *K*α radiation, λ = 1.54187 Å
Hall symbol: -P 2ybc	Cell parameters from 30848 reflections
*a* = 11.6017 (2) Å	θ = 3.6–68.2°
*b* = 12.3381 (2) Å	µ = 1.73 mm^−^^1^
*c* = 16.2825 (3) Å	*T* = 193 K
β = 90.503 (1)°	Block, colorless
*V* = 2330.64 (7) Å^3^	0.30 × 0.20 × 0.10 mm
*Z* = 4	

### Data collection

**Table:**

Rigaku R-AXIS RAPID diffractometer	4266 independent reflections
Radiation source: rotating anode	3197 reflections with *I* > 2σ(*I*)
Graphite monochromator	*R*_int_ = 0.054
Detector resolution: 10.000 pixels mm^-1^	θ_max_ = 68.2°, θ_min_ = 3.8°
ω scans	*h* = −13→13
Absorption correction: numerical (*NUMABS*; Higashi, 1999)	*k* = −14→14
*T*_min_ = 0.625, *T*_max_ = 0.846	*l* = −19→19
40504 measured reflections	

### Refinement

**Table:**

Refinement on *F*^2^	Secondary atom site location: difference Fourier map
Least-squares matrix: full	Hydrogen site location: inferred from neighbouring sites
*R*[*F*^2^ > 2σ(*F*^2^)] = 0.042	H-atom parameters constrained
*wR*(*F*^2^) = 0.131	*w* = 1/[σ^2^(*F*_o_^2^) + (0.0647*P*)^2^ + 0.4811*P*] where *P* = (*F*_o_^2^ + 2*F*_c_^2^)/3
*S* = 1.15	(Δ/σ)_max_ < 0.001
4266 reflections	Δρ_max_ = 0.21 e Å^−^^3^
313 parameters	Δρ_min_ = −0.24 e Å^−^^3^
0 restraints	Extinction correction: *SHELXL97* (Sheldrick, 2008), Fc^*^=kFc[1+0.001xFc^2^λ^3^/sin(2θ)]^-1/4^
Primary atom site location: structure-invariant direct methods	Extinction coefficient: 0.0019 (2)

### Special details

**Table:**

Geometry. All e.s.d.'s (except the e.s.d. in the dihedral angle between two l.s. planes) are estimated using the full covariance matrix. The cell e.s.d.'s are taken into account individually in the estimation of e.s.d.'s in distances, angles and torsion angles; correlations between e.s.d.'s in cell parameters are only used when they are defined by crystal symmetry. An approximate (isotropic) treatment of cell e.s.d.'s is used for estimating e.s.d.'s involving l.s. planes.
Refinement. Refinement of *F*^2^ against ALL reflections. The weighted *R*-factor *wR* and goodness of fit *S* are based on *F*^2^, conventional *R*-factors *R* are based on *F*, with *F* set to zero for negative *F*^2^. The threshold expression of *F*^2^ > σ(*F*^2^) is used only for calculating *R*-factors(gt) *etc*. and is not relevant to the choice of reflections for refinement. *R*-factors based on *F*^2^ are statistically about twice as large as those based on *F*, and *R*- factors based on ALL data will be even larger.

### Fractional atomic coordinates and isotropic or equivalent isotropic displacement parameters (Å^2^)

**Table:**

	*x*	*y*	*z*	*U*_iso_*/*U*_eq_	
Cl1	0.69235 (5)	0.10716 (5)	0.47771 (3)	0.0588 (2)	
O1	0.20277 (12)	0.08172 (12)	0.27478 (9)	0.0481 (4)	
O2	0.33992 (13)	−0.06714 (11)	0.17961 (9)	0.0507 (4)	
O3	−0.02500 (13)	0.23149 (13)	0.17441 (10)	0.0584 (4)	
O4	0.55327 (12)	0.05151 (12)	0.09221 (9)	0.0492 (4)	
C1	0.15846 (17)	0.16470 (15)	0.14692 (12)	0.0393 (5)	
C2	0.06994 (18)	0.23511 (17)	0.12574 (13)	0.0454 (5)	
C3	0.07890 (19)	0.30891 (17)	0.06063 (13)	0.0489 (5)	
H3	0.0164	0.3555	0.0470	0.059*	
C4	0.17854 (19)	0.31268 (17)	0.01738 (13)	0.0480 (5)	
H4	0.1846	0.3618	−0.0273	0.058*	
C5	0.27304 (18)	0.24567 (15)	0.03717 (12)	0.0411 (5)	
C6	0.37374 (19)	0.25238 (17)	−0.01035 (12)	0.0453 (5)	
H6	0.3761	0.3020	−0.0549	0.054*	
C7	0.46715 (18)	0.19001 (17)	0.00576 (12)	0.0459 (5)	
H7	0.5338	0.1948	−0.0275	0.055*	
C8	0.46389 (17)	0.11829 (16)	0.07222 (12)	0.0416 (5)	
C9	0.36732 (17)	0.10735 (15)	0.12160 (12)	0.0379 (4)	
C10	0.26600 (17)	0.17072 (15)	0.10417 (12)	0.0382 (4)	
C11	0.13588 (17)	0.08802 (15)	0.21667 (13)	0.0402 (5)	
C12	0.02723 (16)	0.02178 (15)	0.21450 (12)	0.0389 (4)	
C13	−0.05043 (17)	0.03204 (16)	0.28009 (12)	0.0424 (5)	
C14	−0.15272 (17)	−0.02578 (16)	0.27704 (12)	0.0436 (5)	
H14	−0.2059	−0.0179	0.3207	0.052*	
C15	−0.18025 (17)	−0.09461 (16)	0.21268 (13)	0.0414 (5)	
C16	−0.10099 (17)	−0.10558 (16)	0.14962 (13)	0.0421 (5)	
H16	−0.1179	−0.1536	0.1055	0.050*	
C17	0.00221 (17)	−0.04836 (16)	0.14928 (12)	0.0404 (5)	
C18	0.38091 (16)	0.02322 (15)	0.18818 (12)	0.0394 (5)	
C19	0.45487 (16)	0.04930 (15)	0.26164 (12)	0.0388 (4)	
C20	0.46449 (18)	−0.02682 (16)	0.32407 (12)	0.0449 (5)	
H20	0.4207	−0.0918	0.3211	0.054*	
C21	0.53713 (18)	−0.00905 (17)	0.39053 (13)	0.0476 (5)	
H21	0.5439	−0.0617	0.4329	0.057*	
C22	0.59999 (17)	0.08638 (17)	0.39460 (13)	0.0446 (5)	
C23	0.59021 (17)	0.16476 (17)	0.33401 (12)	0.0442 (5)	
H23	0.6331	0.2302	0.3376	0.053*	
C24	0.51680 (17)	0.14576 (16)	0.26821 (12)	0.0416 (5)	
H24	0.5084	0.1994	0.2267	0.050*	
C25	−0.1238 (2)	0.2937 (2)	0.15477 (17)	0.0650 (7)	
H25A	−0.1048	0.3710	0.1584	0.078*	
H25B	−0.1854	0.2768	0.1935	0.078*	
H25C	−0.1496	0.2765	0.0988	0.078*	
C26	0.66420 (18)	0.0740 (2)	0.05727 (15)	0.0569 (6)	
H26A	0.6843	0.1501	0.0671	0.068*	
H26B	0.6614	0.0602	−0.0020	0.068*	
H26C	0.7224	0.0272	0.0829	0.068*	
C27	−0.0277 (2)	0.1066 (2)	0.35151 (15)	0.0607 (6)	
H27A	−0.0005	0.1767	0.3312	0.073*	
H27B	0.0312	0.0746	0.3876	0.073*	
H27C	−0.0990	0.1170	0.3823	0.073*	
C28	−0.29352 (17)	−0.15531 (17)	0.21101 (14)	0.0465 (5)	
H28A	−0.2807	−0.2295	0.1916	0.056*	
H28B	−0.3477	−0.1185	0.1739	0.056*	
H28C	−0.3256	−0.1573	0.2665	0.056*	
C29	0.08631 (19)	−0.06858 (19)	0.07974 (14)	0.0513 (5)	
H29A	0.0572	−0.1274	0.0448	0.062*	
H29B	0.1616	−0.0889	0.1027	0.062*	
H29C	0.0942	−0.0025	0.0469	0.062*	

### Atomic displacement parameters (Å^2^)

**Table:**

	*U*^11^	*U*^22^	*U*^33^	*U*^12^	*U*^13^	*U*^23^
Cl1	0.0508 (3)	0.0793 (4)	0.0463 (3)	0.0077 (3)	−0.0052 (3)	−0.0046 (3)
O1	0.0416 (8)	0.0590 (9)	0.0436 (9)	−0.0052 (7)	−0.0041 (7)	0.0070 (7)
O2	0.0572 (9)	0.0425 (8)	0.0525 (9)	−0.0051 (7)	−0.0018 (7)	0.0016 (6)
O3	0.0450 (8)	0.0610 (10)	0.0693 (11)	0.0130 (7)	0.0078 (8)	0.0100 (8)
O4	0.0459 (8)	0.0531 (8)	0.0487 (9)	0.0059 (7)	0.0125 (7)	0.0070 (7)
C1	0.0413 (11)	0.0385 (10)	0.0381 (11)	−0.0018 (8)	−0.0014 (9)	−0.0014 (8)
C2	0.0439 (11)	0.0458 (11)	0.0464 (12)	0.0012 (9)	−0.0032 (10)	−0.0028 (9)
C3	0.0529 (13)	0.0435 (11)	0.0502 (13)	0.0031 (10)	−0.0098 (10)	0.0025 (9)
C4	0.0599 (14)	0.0418 (11)	0.0421 (12)	−0.0030 (10)	−0.0105 (10)	0.0039 (9)
C5	0.0487 (11)	0.0386 (10)	0.0360 (11)	−0.0062 (9)	−0.0068 (9)	−0.0005 (8)
C6	0.0552 (13)	0.0460 (11)	0.0346 (11)	−0.0099 (10)	−0.0017 (9)	0.0028 (9)
C7	0.0501 (12)	0.0498 (12)	0.0380 (11)	−0.0078 (10)	0.0043 (9)	−0.0002 (9)
C8	0.0456 (11)	0.0418 (11)	0.0375 (11)	−0.0019 (9)	0.0019 (9)	−0.0014 (8)
C9	0.0410 (10)	0.0393 (10)	0.0336 (10)	−0.0020 (8)	0.0020 (8)	−0.0001 (8)
C10	0.0439 (11)	0.0362 (10)	0.0345 (10)	−0.0054 (8)	−0.0036 (8)	−0.0023 (8)
C11	0.0393 (11)	0.0408 (10)	0.0405 (12)	0.0016 (8)	0.0024 (9)	−0.0011 (8)
C12	0.0370 (10)	0.0415 (10)	0.0381 (11)	0.0017 (8)	−0.0005 (8)	0.0014 (8)
C13	0.0436 (11)	0.0451 (11)	0.0384 (11)	−0.0003 (9)	0.0027 (9)	−0.0040 (9)
C14	0.0419 (11)	0.0477 (11)	0.0414 (11)	−0.0003 (9)	0.0054 (9)	0.0010 (9)
C15	0.0380 (11)	0.0413 (11)	0.0449 (12)	0.0024 (8)	−0.0031 (9)	0.0036 (9)
C16	0.0429 (11)	0.0429 (11)	0.0404 (11)	0.0016 (9)	−0.0041 (9)	−0.0031 (9)
C17	0.0414 (11)	0.0433 (10)	0.0366 (11)	0.0032 (8)	0.0010 (9)	0.0012 (8)
C18	0.0375 (10)	0.0388 (10)	0.0419 (11)	0.0021 (8)	0.0061 (9)	0.0003 (8)
C19	0.0372 (10)	0.0410 (10)	0.0382 (11)	0.0038 (8)	0.0063 (8)	0.0013 (8)
C20	0.0496 (12)	0.0416 (11)	0.0437 (12)	0.0002 (9)	0.0054 (10)	0.0058 (9)
C21	0.0534 (12)	0.0499 (12)	0.0394 (12)	0.0087 (10)	0.0028 (10)	0.0069 (9)
C22	0.0404 (11)	0.0547 (12)	0.0387 (12)	0.0088 (9)	0.0043 (9)	−0.0031 (9)
C23	0.0420 (11)	0.0475 (11)	0.0431 (12)	−0.0004 (9)	0.0061 (9)	−0.0040 (9)
C24	0.0433 (11)	0.0425 (11)	0.0391 (11)	0.0034 (9)	0.0052 (9)	0.0038 (9)
C25	0.0433 (13)	0.0658 (15)	0.0857 (19)	0.0084 (11)	−0.0070 (12)	−0.0008 (13)
C26	0.0432 (12)	0.0672 (15)	0.0605 (15)	−0.0001 (10)	0.0090 (11)	0.0034 (11)
C27	0.0555 (14)	0.0743 (16)	0.0526 (14)	−0.0118 (12)	0.0093 (11)	−0.0193 (12)
C28	0.0417 (11)	0.0487 (12)	0.0491 (13)	−0.0067 (9)	−0.0024 (9)	0.0026 (9)
C29	0.0477 (12)	0.0623 (14)	0.0439 (13)	−0.0021 (10)	0.0040 (10)	−0.0099 (10)

### Geometric parameters (Å, º)

**Table:**

Cl1—C22	1.738 (2)	C15—C16	1.391 (3)
O1—C11	1.221 (2)	C15—C28	1.513 (3)
O2—C18	1.220 (2)	C16—C17	1.390 (3)
O3—C2	1.363 (2)	C16—H16	0.9500
O3—C25	1.414 (3)	C17—C29	1.522 (3)
O4—C8	1.362 (2)	C18—C19	1.501 (3)
O4—C26	1.439 (2)	C19—C20	1.388 (3)
C1—C2	1.386 (3)	C19—C24	1.394 (3)
C1—C10	1.436 (3)	C20—C21	1.383 (3)
C1—C11	1.503 (3)	C20—H20	0.9500
C2—C3	1.402 (3)	C21—C22	1.386 (3)
C3—C4	1.360 (3)	C21—H21	0.9500
C3—H3	0.9500	C22—C23	1.385 (3)
C4—C5	1.408 (3)	C23—C24	1.383 (3)
C4—H4	0.9500	C23—H23	0.9500
C5—C6	1.409 (3)	C24—H24	0.9500
C5—C10	1.433 (3)	C25—H25A	0.9800
C6—C7	1.353 (3)	C25—H25B	0.9800
C6—H6	0.9500	C25—H25C	0.9800
C7—C8	1.399 (3)	C26—H26A	0.9800
C7—H7	0.9500	C26—H26B	0.9800
C8—C9	1.391 (3)	C26—H26C	0.9800
C9—C10	1.438 (3)	C27—H27A	0.9800
C9—C18	1.508 (3)	C27—H27B	0.9800
C11—C12	1.502 (3)	C27—H27C	0.9800
C12—C17	1.398 (3)	C28—H28A	0.9800
C12—C13	1.409 (3)	C28—H28B	0.9800
C13—C14	1.385 (3)	C28—H28C	0.9800
C13—C27	1.504 (3)	C29—H29A	0.9800
C14—C15	1.384 (3)	C29—H29B	0.9800
C14—H14	0.9500	C29—H29C	0.9800
			
C2—O3—C25	120.54 (18)	C12—C17—C29	122.43 (18)
C8—O4—C26	118.07 (16)	O2—C18—C19	120.45 (18)
C2—C1—C10	119.46 (18)	O2—C18—C9	120.58 (18)
C2—C1—C11	116.67 (17)	C19—C18—C9	118.73 (16)
C10—C1—C11	123.84 (17)	C20—C19—C24	118.92 (19)
O3—C2—C1	115.81 (18)	C20—C19—C18	118.71 (18)
O3—C2—C3	121.77 (19)	C24—C19—C18	122.35 (18)
C1—C2—C3	122.37 (19)	C21—C20—C19	120.7 (2)
C4—C3—C2	118.9 (2)	C21—C20—H20	119.7
C4—C3—H3	120.6	C19—C20—H20	119.7
C2—C3—H3	120.6	C20—C21—C22	119.26 (19)
C3—C4—C5	121.7 (2)	C20—C21—H21	120.4
C3—C4—H4	119.2	C22—C21—H21	120.4
C5—C4—H4	119.2	C23—C22—C21	121.3 (2)
C4—C5—C6	119.17 (19)	C23—C22—Cl1	119.84 (17)
C4—C5—C10	120.24 (18)	C21—C22—Cl1	118.89 (17)
C6—C5—C10	120.59 (19)	C24—C23—C22	118.64 (19)
C7—C6—C5	121.77 (19)	C24—C23—H23	120.7
C7—C6—H6	119.1	C22—C23—H23	120.7
C5—C6—H6	119.1	C23—C24—C19	121.19 (19)
C6—C7—C8	118.87 (19)	C23—C24—H24	119.4
C6—C7—H7	120.6	C19—C24—H24	119.4
C8—C7—H7	120.6	O3—C25—H25A	109.5
O4—C8—C9	114.75 (17)	O3—C25—H25B	109.5
O4—C8—C7	122.82 (17)	H25A—C25—H25B	109.5
C9—C8—C7	122.40 (19)	O3—C25—H25C	109.5
C8—C9—C10	119.62 (18)	H25A—C25—H25C	109.5
C8—C9—C18	113.73 (17)	H25B—C25—H25C	109.5
C10—C9—C18	126.60 (16)	O4—C26—H26A	109.5
C5—C10—C1	117.25 (18)	O4—C26—H26B	109.5
C5—C10—C9	116.69 (17)	H26A—C26—H26B	109.5
C1—C10—C9	126.05 (17)	O4—C26—H26C	109.5
O1—C11—C12	120.73 (17)	H26A—C26—H26C	109.5
O1—C11—C1	120.79 (18)	H26B—C26—H26C	109.5
C12—C11—C1	118.45 (18)	C13—C27—H27A	109.5
C17—C12—C13	120.09 (18)	C13—C27—H27B	109.5
C17—C12—C11	121.53 (17)	H27A—C27—H27B	109.5
C13—C12—C11	118.38 (17)	C13—C27—H27C	109.5
C14—C13—C12	118.68 (18)	H27A—C27—H27C	109.5
C14—C13—C27	119.13 (18)	H27B—C27—H27C	109.5
C12—C13—C27	122.15 (18)	C15—C28—H28A	109.5
C15—C14—C13	122.35 (19)	C15—C28—H28B	109.5
C15—C14—H14	118.8	H28A—C28—H28B	109.5
C13—C14—H14	118.8	C15—C28—H28C	109.5
C14—C15—C16	117.96 (18)	H28A—C28—H28C	109.5
C14—C15—C28	120.80 (18)	H28B—C28—H28C	109.5
C16—C15—C28	121.23 (19)	C17—C29—H29A	109.5
C17—C16—C15	121.92 (19)	C17—C29—H29B	109.5
C17—C16—H16	119.0	H29A—C29—H29B	109.5
C15—C16—H16	119.0	C17—C29—H29C	109.5
C16—C17—C12	118.97 (18)	H29A—C29—H29C	109.5
C16—C17—C29	118.54 (18)	H29B—C29—H29C	109.5
			
C25—O3—C2—C1	−175.14 (19)	O1—C11—C12—C17	−123.9 (2)
C25—O3—C2—C3	7.5 (3)	C1—C11—C12—C17	58.0 (3)
C10—C1—C2—O3	−173.78 (17)	O1—C11—C12—C13	56.4 (3)
C11—C1—C2—O3	4.2 (3)	C1—C11—C12—C13	−121.7 (2)
C10—C1—C2—C3	3.6 (3)	C17—C12—C13—C14	−2.0 (3)
C11—C1—C2—C3	−178.48 (19)	C11—C12—C13—C14	177.67 (18)
O3—C2—C3—C4	176.24 (19)	C17—C12—C13—C27	−179.8 (2)
C1—C2—C3—C4	−0.9 (3)	C11—C12—C13—C27	−0.1 (3)
C2—C3—C4—C5	−1.0 (3)	C12—C13—C14—C15	1.2 (3)
C3—C4—C5—C6	179.39 (19)	C27—C13—C14—C15	179.0 (2)
C3—C4—C5—C10	0.2 (3)	C13—C14—C15—C16	0.4 (3)
C4—C5—C6—C7	−179.97 (19)	C13—C14—C15—C28	−179.21 (19)
C10—C5—C6—C7	−0.8 (3)	C14—C15—C16—C17	−1.2 (3)
C5—C6—C7—C8	−1.0 (3)	C28—C15—C16—C17	178.41 (19)
C26—O4—C8—C9	−166.11 (18)	C15—C16—C17—C12	0.4 (3)
C26—O4—C8—C7	15.8 (3)	C15—C16—C17—C29	177.52 (19)
C6—C7—C8—O4	179.09 (18)	C13—C12—C17—C16	1.3 (3)
C6—C7—C8—C9	1.1 (3)	C11—C12—C17—C16	−178.42 (18)
O4—C8—C9—C10	−177.55 (17)	C13—C12—C17—C29	−175.75 (19)
C7—C8—C9—C10	0.6 (3)	C11—C12—C17—C29	4.5 (3)
O4—C8—C9—C18	0.2 (3)	C8—C9—C18—O2	−99.7 (2)
C7—C8—C9—C18	178.27 (18)	C10—C9—C18—O2	77.8 (3)
C4—C5—C10—C1	2.4 (3)	C8—C9—C18—C19	74.7 (2)
C6—C5—C10—C1	−176.82 (18)	C10—C9—C18—C19	−107.8 (2)
C4—C5—C10—C9	−178.44 (18)	O2—C18—C19—C20	−7.5 (3)
C6—C5—C10—C9	2.4 (3)	C9—C18—C19—C20	178.04 (17)
C2—C1—C10—C5	−4.1 (3)	O2—C18—C19—C24	170.53 (18)
C11—C1—C10—C5	178.04 (17)	C9—C18—C19—C24	−3.9 (3)
C2—C1—C10—C9	176.74 (18)	C24—C19—C20—C21	−2.1 (3)
C11—C1—C10—C9	−1.1 (3)	C18—C19—C20—C21	176.06 (17)
C8—C9—C10—C5	−2.2 (3)	C19—C20—C21—C22	0.5 (3)
C18—C9—C10—C5	−179.63 (18)	C20—C21—C22—C23	0.9 (3)
C8—C9—C10—C1	176.87 (18)	C20—C21—C22—Cl1	−179.21 (15)
C18—C9—C10—C1	−0.5 (3)	C21—C22—C23—C24	−0.6 (3)
C2—C1—C11—O1	−127.5 (2)	Cl1—C22—C23—C24	179.47 (14)
C10—C1—C11—O1	50.4 (3)	C22—C23—C24—C19	−1.0 (3)
C2—C1—C11—C12	50.6 (2)	C20—C19—C24—C23	2.3 (3)
C10—C1—C11—C12	−131.50 (19)	C18—C19—C24—C23	−175.70 (17)

### Hydrogen-bond geometry (Å, º)

**Table:**

*D*—H···*A*	*D*—H	H···*A*	*D*···*A*	*D*—H···*A*
C23—H23···O2^i^	0.95	2.54	3.413 (2)	154
C28—H28*A*···O1^ii^	0.98	2.56	3.418 (3)	147
C29—H29*B*···O2	0.98	2.42	3.349 (3)	157

Symmetry codes: (i) −*x*+1, *y*+1/2, −*z*+1/2; (ii) −*x*, *y*−1/2, −*z*+1/2.
